# Recent Advances in Curcumin-Based Combination Nanomedicines for Cancer Therapy

**DOI:** 10.3390/jfb14080408

**Published:** 2023-08-02

**Authors:** Amir R. Afshari, Mehdi Sanati, Prashant Kesharwani, Amirhossein Sahebkar

**Affiliations:** 1 Department of Physiology and Pharmacology, Faculty of Medicine, North Khorasan University of Medical Sciences, Bojnurd, Iran; 2 Department of Pharmacology and Toxicology, Faculty of Pharmacy, Birjand University of Medical Sciences, Birjand, Iran; 3 Experimental and Animal Study Center, Birjand University of Medical Sciences, Birjand, Iran; 4 Department of Pharmaceutics, School of Pharmaceutical Education and Research, Jamia Hamdard, New Delhi 110062, India; 5 Department of Pharmacology, Saveetha Dental College, Saveetha Institute of Medical and Technical Sciences, Saveetha University, Chennai 602105, India; 6 Biotechnology Research Center, Pharmaceutical Technology Institute, Mashhad University of Medical Sciences, Mashhad, Iran; 7 Applied Biomedical Research Center, Mashhad University of Medical Sciences, Mashhad, Iran

**Keywords:** curcumin, cancer, combination therapy, drug delivery, drug resistance

## Abstract

Standard cancer chemotherapeutics often produce significant adverse effects and eventually lose their effectiveness due to the emergence of resistance mechanisms. As a result, patients with malignant tumors experience a poor quality of life and a short lifespan. Thus, combination medication regimens provide various advantages, including increased success rate, fewer side effects, and fewer occurrences of resistance. Curcumin (Cur), a potential phytochemical from turmeric, when coupled with traditional chemotherapeutics, has been established to improve the effectiveness of cancer treatment in clinical and preclinical investigations. Cur not only exerts multiple mechanisms resulting in apoptotic cancer cell death but also reduces the resistance to standard chemotherapy drugs, mainly through downregulating the multi-drug resistance (MDR) cargoes. Recent reports showed the beneficial outcomes of Cur combination with many chemotherapeutics in various malignancies. Nevertheless, owing to the limited bioavailability, devising co-delivery strategies for Cur and conventional pharmaceuticals appears to be required for clinical settings. This review summarized various Cur combinations with standard treatments as cancer therapeutics.

## 1. Introduction

Combination therapy is now a leading aspect of cancer therapy. In comparison to the monotherapy method, combining anti-cancer medications improves effectiveness due to targeting important pathways synergistically or additively. This strategy can potentially diminish drug resistance whilst also offering therapeutic anti-cancer advantages, e.g., halting mitotically active cells, lowering cancer stem cell populations, and causing apoptosis in cancerous cells [1]. The five-year survival rate of most metastatic malignancies is still relatively poor, and launching a unique anti-cancer treatment is both highly costly and exceedingly time-demanding [2,3,4]. A novel approach that focuses on survival routes and provides cost-effective and high-quality outcomes is thus urgently needed.

Chemotherapy medicines, e.g., docetaxel (DTX), irinotecan, 5-fluorouracil (5-FU), cisplatin, and paclitaxel (PTX), are utilized in treating diverse cancers; nevertheless, their efficacies are confined, owing to the emergence of drug resistance, inducing non-selective toxicity and producing significant adverse reactions [5]. It is expected that the efficiency of pharmacological therapy will improve when combined with treatments that use phytochemicals, and the unwanted effects will become less severe as a result [6,7,8]. For instance, the findings showed that curcumin (Cur, an active ingredient from *Curcuma longa* with multiple biological effects [9,10,11,12,13,14,15,16,17]), in combination with chemotherapeutics, had a wider range of anti-cancer action and a lower risk of severe side effects than standard chemotherapy medications [18,19]. In corroboration with the preclinical data, over the past decade, more than 20 clinical trials have assessed the efficacy and safety of Cur, alone or in combination with other chemotherapeutic agents, for treating various malignancies. The emerging clinical data validated the remarkable potential of Cur in cancer therapy [20,21]. More interestingly, with the progress of nanotechnology, targeted drug delivery systems have been designed to promote the bioavailability of relatively water-insoluble Cur and the co-delivery of Cur and conventional chemotherapeutics to the tumor site [22,23]. This review recapitulated the advancements in Cur and standard chemotherapy drugs combination therapy in various malignancies.

## 2. Curcumin-Based Combination Anti-Cancer Drug Delivery Systems against Colorectal Cancer

Colorectal cancer (CRC) is the third cause of cancer mortality worldwide. Although 5-FU and oxaliplatin are the main treatments, they are commonly used in chemotherapy regimens to suppress CRC progression. Unfortunately, CRC patients who are given 5-FU frequently experience multi-drug resistance (MDR). The issue is partially solved by raising the amount of 5-FU; however, excessive doses would cause significant toxicities; therefore, the therapy must be halted [24]. These patients may benefit from the combination of 5-FU and Cur, which has been shown to be helpful in overcoming drug resistance and decreasing 5-FU cytotoxicity. For example, Cur was demonstrated to increase 5-FU chemosensitivity and reverse mismatch repair (MMR)-deficient CRC cell chemoresistance [25]. Furthermore, Cur boosted 5-FU-mediated cleavage of pro-apoptotic proteins (e.g., caspases, peroxisome proliferator-activated receptor (PARP), and Bax) and downregulated Bcl-xL and cyclin D1 proteins in resistant CRC cells. Interestingly, Cur was found to reverse the 5-FU-activated nuclear factor kappa B (NF-κB)/phosphoinositide 3-kinase (PI3K)/Src signaling-mediated MDR development. As proof of concept, repressing IκB kinase (IKK) function and IκBα phosphorylation abolished the Cur chemosensitizing impact [26,27]. Similarly, Cur therapy was recently demonstrated to improve the CRC xenografts’ sensitivity to 5-FU cytotoxicity [28]. Moreover, Meiyanto et al., in their investigation on CRC cells, showed that 5-FU cytotoxicity increased when combined with PGV-1 (a Cur derivative with advanced cytotoxicity for cancer cells), and more profound growth inhibition was observed compared to each treatment. Apart from causing cell cycle arrest, PGV-1 reduced NF-κB activation and lowered cyclooxygenase-2 (COX-2) expression, an NF-κB downstream protein, indicating that PGV-1 might be a beneficial agent for cancer combination therapy [29]. Accordingly, it is possible to conclude from these findings that pretreatment with Cur or its derivatives is able to enhance sensitivity to 5-FU and invert MDR in resistant CRC cells by inhibiting NF-κB. Consistently, Cur was demonstrated to lower the chemoresistance of CRC cells by decreasing the expression of multidrug resistance protein 1 (MRP1) and P-glycoprotein (P-gp), two proteins involved in drug resistance [30]. 

As a result of chemosensitizing, the Cur and 5-FU co-administration has shown promising anti-cancer effects. For example, a combination of Cur (50 mg/kg) and 5-FU (20 mg/kg) suppressed epithelial-to-mesenchymal transition (EMT) and proliferation in resistant CRC cells. As expected, Cur also enhanced the 5-FU ability to prevent tumor development in a xenografted mice model [31]. In line with these findings, Cur was efficient in hindering cell multiplication, provoking apoptosis, and blocking G0/G1 phase in 5-FU-treated HCT-116 cells. Notably, Cur reduced the expression of ten-eleven translocation 1 (TET1) and naked cuticle homolog (NKD2), indicating that Cur might potentiate the impact of 5-FU on HCT-116 cells by restraining the TET1/NKD2/Wnt signaling and inhibiting the EMT [32]. Oxaliplatin resistance is also a real challenge in treating CRC that may be attributed to the transforming growth factor beta (TGF-β)/Smad signaling-induced EMT. In Yin et al.’s study, it was found that combining oxaliplatin with Cur reversed oxaliplatin resistance in HCT116 cells by affecting the TGF-β/Smad signaling and EMT in vitro and in vivo. The combination therapy also attenuated the expressions of p65 and Bcl-2 while enhancing the status of cleaved caspase-3 [33]. Accordingly, combining Cur with standard chemotherapeutics may be a suitable strategy to improve CRC patient prognosis. Intriguingly, recruiting nanotechnology for designing drug delivery systems significantly increases the beneficial outcomes of such combination therapies. For instance, incorporating Cur and 5-FU into thiolate chitosan nanoparticles was indicated to augment their anti-cancer impacts on CRC cells and increase the bioavailability of drugs compared to the non-formulated combination [34,35]. In another work by Sesarman et al., the anti-tumoral action of polyethylene glycol (PEG)ylated long-circulating nanoliposomes (LCL) co-delivering Cur and doxorubicin (DOX) on murine CRC cells (C26) was investigated. PEGylated LCL-Cur-DOX exhibited powerful antiproliferative impacts on C26 cells via inhibiting the generation of angiogenic/inflammatory proteins in an NF-κB-dependent way [36].

In recent years, FOLFOX chemotherapy (i.e., a combination drug regimen containing folinic acid, 5-FU, and oxaliplatin) remains the mainstay of CRC treatment, although they had minimal effectiveness. This may be attributed partly to the cancer stem cells (CSCs) being resistant to standard therapy. Specifically, Yu et al. investigated whether Cur or Cur plus FOLFOX chemotherapy might eradicate colon CSCs. It was shown that Cur, alone or in combination with FOLFOX, significantly reduced CSCs in the FOLFOX-surviving CRC cell population, as demonstrated by the lower expression of CD44 and CD166 and epidermal growth factor receptor (EGFR) and by the reduced capacity of cells to create anchorage-dependent colonies [37]. In another study, researchers found that Cur and FOLFOX performed together to boost cell growth inhibition in HCT-116 and HT-29 cells. The treatment inactivated EGFR, human epidermal growth factor receptor 2 (HER2), insulin-like growth factor type-1 receptor (IGF-1R), and AKT and reduced the expressions of COX-2 and cyclin-D1 in order to limit chemo-surviving cell formation [38]. Intriguingly, a phase I clinical trial in CRC patients with inoperable liver metastases revealed that Cur is a safe and well-tolerated adjuvant to FOLFOX treatment at dosages up to 2 g/day and may provide added benefits in subsets of patients [39]. Similarly, a randomized phase IIa trial found that Cur plus FOLFOX chemotherapy is safe and endurable in patients with metastatic CRC cancer [40].

## 3. Curcumin-Based Combination Anti-Cancer Drug Delivery Systems against Breast Cancer

For treating breast cancer, the primary health issue confronting women, 5-FU alone or combined with conventional chemotherapy medicines has extensively been employed. Intriguingly, the viability of breast cancer cells was considerably reduced through apoptosis induction when Cur (10 µM) and 5-FU (10 µM) were combined in vitro [41]. Furthermore, silk fibroin (SF) nanoparticles loaded with 5-FU and Cur were shown to be more efficient in killing breast cancer cells in vitro and in vivo than free Cur and 5-FU, mainly through provoking cellular ROS generation [42]. As another breast cancer treatment, PTX is a clinical drug that has anti-cancer action in diverse cancer cells. In this regard, Calaf et al. evaluated cell death induced by Cur and PTX alone and in combination in MCF-7 and MDA-MB-234 breast cancer cells. The findings suggested that both Cur and PTX were responsible for inducing apoptosis and necrosis. The apoptotic death was more profoundly induced by the Cur and PTX combination compared to either chemical alone. Consequently, using a multi-drug approach in chemotherapy may be helpful in treating breast cancer [43]. Another experiment engaged GANT61, a hexahydro pyrimidine product that selectively hindered GLI transcription factors in the Hedgehog signaling pathway when combined with Cur. The agents were co-delivered using polymeric nanoparticles in order to elicit a more significant anti-tumor impact against the heterogeneous MCF-7 breast cancer cells. The outcomes indicated that nanoparticles generated lethal effects at a mid-minimal dose, pursued by cell death through autophagy and apoptosis, a decrease in the target proteins expression, and a compromise of the self-renewal capacity of CSCs [44]. It is also worth mentioning that Cur- and methotrexate-co-encapsulated polylactic-co-glycolic acid (PLGA) nanoparticles, as a possible treatment for breast cancer, displayed much greater cytotoxicity than free methotrexate, Cur, or even their solo-loaded formulations. Co-delivery of methotrexate and Cur was shown to generate a synergistic effect on the advancement of breast cancer cells. Regarding the desired in vitro characteristics, it seems that the generated formulation is a suitable option for future in vivo investigations [45]. Furthermore, a targeted delivery system based on a Ni/Ta core–shell metal–organic framework coated with folic acid-activated chitosan nanoparticles exhibited a synergistic impact in suppressing breast cancer cells via the co-delivery of DOX and Cur and sensitizing breast cancer cells to DOX [46]. Hence, the dual-drug loaded nanoparticles provide a fresh viewpoint on assisting current anti-cancer nanomedicine treatments in efficiently targeting a heterogeneous tumor mass.

## 4. Curcumin-Based Combination Anti-Cancer Drug Delivery Systems against Prostate Cancer

Prostate cancer is the most prevalent diagnosis among men. It has been shown that using a combined chemotherapy plan for prostate cancer is a successful method. DTX has been extensively utilized in treating metastatic-castration-resistant prostate cancer for many years; nevertheless, extended treatment with this medication may induce significant toxicity in individuals [47]. In a study carried out by Banerjee et al., it was discovered that treating prostate cancer cells with Cur (20 µM) and DTX (10 nM) remarkably suppressed the multiplication of cells and provoked apoptosis in comparison to the treatment with each medication. Cur was demonstrated to improve the efficiency of DTX in PC-3 cells via modulating COX-2, p53, NF-κB, phospho-Akt, PI3K, and receptor tyrosine kinase (RTK) expression or function [48]. Of note, a pilot phase II clinical study indicated that the combination of Cur and DTX was well tolerated in prostate cancer patients [49]. Cur has also been shown to improve the sensitivity of prostate cancer cells to PTX through targeting mitogen and stress-activated kinase 1 (MSK1) and insulin receptor substrate-1 (IRS-1) signaling. The combination remarkably augmented apoptosis and reduced the expression of P-gp in CD44^+^ cells in comparison to Cur or PTX [50]. Furthermore, the combination of Cur and metformin displayed a synergistic impact in triggering apoptosis in LNCaP prostate cancer cells, as indicated by suppressing mTOR signaling, upregulating Bax and PUMA, and reducing the Bcl-2 expression [51]. Accordingly, combining Cur with chemotherapeutics for prostate cancer patients might be a viable treatment plan to reduce adverse effects, overcome drug resistance, and improve therapeutic outcomes. Moreover, nanoparticles such as lipid-polymer hybrid nanoparticles (LPNs) provide considerable benefits for combined prostate cancer treatment. In this regard, Chen et al. originated an aptamer-conjugated ligand and produced aptamer-functionalized Cur and cabazitaxel co-delivered LPNs (APT-CUR/CTX-LPNs). The nano-drug delivery system demonstrated noticeable cancer cell targeting, significant tumor penetration, and outstanding tumor suppression. These findings revealed that novel nanocarriers are promising tools for a synergistic combination treatment of prostate cancer [52].

## 5. Curcumin-Based Combination Anti-Cancer Drug Delivery Systems against Other Cancers

Cur-based combination therapy provides significant promise in diverse cancers (Table 1). The outcomes of the study by Fratantonio et al. confirmed that the antiproliferative function of PTX against murine glioma C6 cells was improved by Cur, as indicated by reducing clonogenic capacity, halting the cell cycle, provoking apoptosis, hindering NF-κB, and generating ROS. Additionally, the combination boosted the levels of p53 and p21, strengthening the antiproliferative impacts. These findings showed that Cur and PTX worked together to improve anti-glioma effectiveness in vitro, which might lead to lower doses of cytotoxic treatment and lessen adverse reactions [53]. Furthermore, the combination of Cur (10 µM) and FOLFOX chemotherapy caused the synergistic anti-tumor effect in BGC-823 gastric cancer cells, compared to each treatment, by reducing the Bcl-2 transcription and protein expression and increasing the Bax and caspases-3, 8, and 9 levels. Moreso, combining Cur and FOLFOX inhibited BGC-823 xenograft tumor development more effectively than 5-FU, oxaliplatin, or Cur alone [54]. Another experiment also found that the combination of 5-FU (50 µM) and Cur (25 µM) augmented cytotoxicity against AGS gastric cancer cells by interfering with the NF-κB signaling pathway and decreasing the COX-2 production. These findings recommend that Cur is probably modulating inflammatory cytokine production to promote the chemosensitivity of gastric cancer cells [55]. 

**Figure 1:**
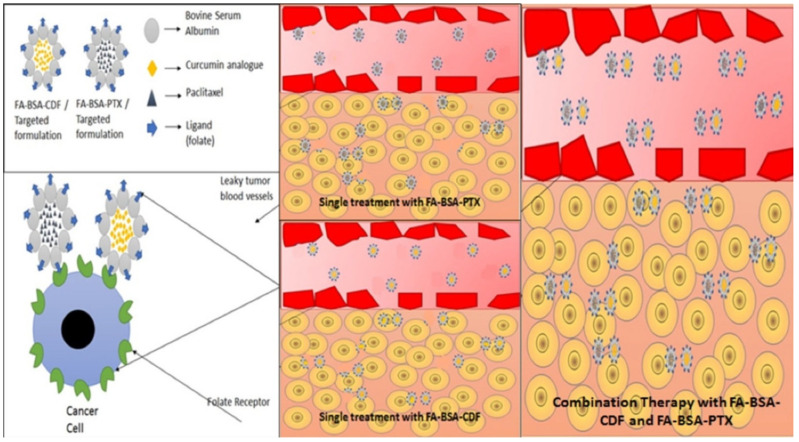
Mechanism of receptor-mediated endocytosis for tumor cell-targeted delivery and internalization of combination drugs. The albumin base nanoparticles co-loaded with drugs can take advantage of leaky tumor vasculature or the EPR effect to accumulate in tumor tissues, followed by folate receptor-mediated endocytosis into tumor cells overexpressing the target. Reprinted with permission from Ref. [68]. Copyright 2018 Elsevier.

The FDA recently authorized the first-line therapy for advanced metastatic pancreatic, lung, and breast malignancies using PTX encapsulated in albumin (Abraxane^®^), currently being administered as a component of combination treatment regimens. In addition, difluorinated Cur, often known as CDF, is an innovative and powerful synthetic Cur analog being researched for various malignancies, such as pancreatic, liver, ovarian, and breast cancers. Gawde et al. encapsulated hydrophobic PTX and CDF separately in folic acid-decorated bovine serum albumin (BSA) nanoparticles, which they referred to as FA-BSA-PTX and FA-BSA-CDF, respectively. The goal was to increase the bioavailability and targeting capacities of the compounds. According to their findings, the combination of FA-BSA-CDF and FA-BSA-PTX generated a synergistic anti-cancer effect, probably owing to folate receptor-mediated targeted uptake of nanoparticles by cancerous cells as well as apoptosis activation. These outcomes indicated that the mentioned nanomedicine platform might be used to develop an effective combination treatment for the most common types of gynecological tumors, such as ovarian and cervical cancer [68] (Figure 1). Co-loaded nanoliposomes containing cisplatin and Cur were developed by Cheng et al. to overcome the dismal clinical outcomes of cisplatin monotherapy. During the treatment of hepatocellular carcinoma (HCC) cells, the liposomal cisplatin/Cur formulation displayed the highest anti-tumor efficacy and also enhanced the intracellular ROS levels, providing an attractive strategy to attain a synergistic effect for the treatment of HCC [69]. Hong et al. have developed uPAR-targeting, peptide-decorated, pH-sensitive, Cur- and DOX-loaded (U11-DOX/Cur) nanoparticles to attenuate the severe adverse effects and MDR of DOX treatment. The chemotherapeutic activity of nanoparticles was assessed in lung cancer in vitro and in vivo. U11-DOX/Cur nanoparticles demonstrated a powerful anti-cancer impact and a precise tumor tissue accumulation efficiency in vitro. Furthermore, the U11-DOX/Cur nanoparticles reduced tumor development to 85% in vivo, indicating that these nanoparticles had good potential for combined lung cancer therapy [70]. Moreover, Guo et al. discovered that loading DOX and Cur into polypeptide nanocarriers provided high anti-lymphoma effects with low toxicity [71]. Accordingly, designing nano-targeting platforms for the co-delivery of Cur and standard chemotherapeutics have the potential to revolutionize the therapeutic outcomes of cancer chemotherapy. 

As mentioned, tumor MDR has mostly remained the cause of therapeutic cancer failures. It is a significant issue in treating tumors by chemical therapy or by techniques of surgical intervention. Unfortunately, more than 70% of patients with ovarian cancer are initially resistant to the standard medications, which leads to the recurrence of the disease [72]. Numerous reports revealed that overcoming MDR may minimize the risk of chemotherapy failure, which has a significant therapeutic benefit. Therefore, MDR reversal has emerged as a key area of interest in developing new chemotherapy medicines [73]. With this in mind, Liu et al. revealed that PTX and Cur incorporated into PLGA-phospholipid nanoparticles had increased solubility and stability and a delayed release pattern. As a result of improved drug delivery to the target site, Cur significantly enhanced the intracellular concentration of PTX in cancerous cells to increase its anti-cancer efficacy. This was because Cur dramatically lowers the P-gp level in drug-resistant ovarian cancer cells. Accordingly, the dual drug-loaded PLGA phospholipid nanohybrids were capable of defeating MDR and boosting the efficacy of chemotherapy medications [74]. Recently, a “core–shell” polymeric nanoparticle-mediated Cur and PTX co-delivery platform originated to reduce adverse effects, invert chemoresistance, and enhance the effectiveness of PTX in ovarian cancer. On chemosensitive human ovarian cancer cells (SKOV3) and their MDR variant (SKOV3-TR30), nanocarrier-mediated co-delivery of Cur and PTX caused synergistic anti-cancer impacts in vitro and in vivo. The action of nanosystems in impeding the P-gp-mediated efflux and inhibiting the migration of tumor cells, along with Cur function in reversing the P-gp-induced resistance to PTX, is suggested as the mechanism responsible for reversing drug resistance. It is important to point out that the therapy did not result in a considerable amount of toxicity being caused to the uterus or the ovaries, as shown by macroscopic and microscopic examinations. As a potential technique for treating ovarian cancer, co-delivery of Cur and PTX using targeted delivery nanosystems may boost the anti-tumor activity without raising adverse events [75].

It is noteworthy to mention that Cur has been utilized as a photosensitizer in the context of photodynamic therapy (PDT) for a wide range of malignancies [76,77,78]. PDT is an innovative method harnessing light-sensitive agents and a light source to eliminate abnormal cells by inducing oxidative stress. When combined with nanotechnology, PDT confers more effective and tailored therapeutic impacts [79]. The study by Zhang et al. investigated the combined chemotherapeutic and photosensitizing effects of Cur on melanoma cancer cells. The curcumin-loaded nanoparticles were demonstrated to effectively decrease the expression of hypoxia-inducible factor 1-alpha (HIF-1α) and deplete glutathione, thereby enhancing the vulnerability of cancer cells to PDT. During laser irradiation, Cur exhibited photosensitizing properties, leading to the generation of ROS and the subsequent eradication of B16F10 cells [80]. In a similar context, a recent study examined the potential of curcumin- and cisplatin-loaded mesoporous silica nanoparticles for synergistic chemo-PDT against drug-resistant human uterine sarcoma cells. The hybrid nanocomposites successfully transported Cur and cisplatin into the target cells, where the presence of Cur photosensitizer also increased cellular ROS levels under light irradiation. The integrated approach yielded a remarkable synergistic anti-cancer outcome in eradicating resistant cancer cells [81]. 

Intriguingly, Cur-loaded nanoparticles may be modified in a way that enables the invention of imaging-guided chemotherapeutics. A study by Liu et al. reported that applying a poly-dopamine coating on Cur-loaded 2D nanosheets resulted in the acquisition of multimodal imaging capabilities, facilitating the noninvasive visualization of distribution profiles specifically within the tumor region. In vivo experiments confirmed that the nanoparticles provided imaging-guided tumor chemo-PDT under laser irradiation, with minimal toxicity to surrounding normal tissues [82]. In a further endeavor, Cur-loaded, gadolinium-doped hollow silica nanospheres were engineered to enable magnetic resonance imaging (MRI)-guided synergistic cancer sonodynamic-chemotherapy. Given its substantially greater tissue penetration depth, sonodynamic therapy (SDT) exhibits substantial benefits over PDT. The delivery platforms leveraged the pH-responsive destruction and ultrasound-triggered drug release mechanisms. Moreover, the liberation of gadolinium ions or oligomers during degradation acts as a highly efficient contrast agent for MRI, facilitating the precise direction of cancer treatment. These nanoparticles exhibited an impressive tumor growth inhibition rate of approximately 85.6% when subjected to ultrasound irradiation, primarily attributed to the synergistic effect of sonodynamic-chemotherapy [83]. Sheng et al. employed an innovative strategy to achieve a multiplex imaging-guided programmed delivery of DOX and Cur using a self-fluorescent nanoparticles/hydrogel system composed of PEG and polycaprolactone (PCL) polymer. With the devised platform, DOX and CUR could be administered in a controlled fashion over time, which could be tracked in real-time, in vitro and in vivo [84]. 

Because of their synergistically enhanced apoptotic death of cancer cells through stimulating multiple signaling pathways, combinations of natural agents have garnered much interest in cancer therapy. However, the hydrophobic character of most biological substances, along with their poor bioavailability and limited cellular absorption, severely limited their therapeutic use [85,86]. As a solution, originating delivery systems increasing phytochemicals pharmacokinetics and delivery might improve their clinical anti-cancer impacts [87]. For instance, a nanoliposomal TriCurin (Cur, epicatechin gallate, and resveratrol) induced p53 function in cultured GL261 murine GBM cells to provoke apoptosis of GBM and GBM stem cells in vitro [88]. In Table 2, we demonstrated some potential combinational-regimen-based Cur with other natural products in various cancer cells.

## 6. Conclusions

Combination therapy is one of the solutions for preventing the emergence of drug resistance and severe adverse effects, as well as improving the effectiveness of treatments for malignancies. With this in mind, numerous experiments examined the potential of phytochemicals as adjuvants to standard chemotherapy protocols and found encouraging results. Cur is one such compound that possesses apoptotic effects and suppresses multi-drug resistance mechanisms in cancerous cells, promoting the anti-cancer impacts of chemotherapy drugs and attenuating the likelihood of drug resistance. Furthermore, nano-drug carriers co-delivering Cur and chemotherapeutics to the site of action add value to the treatment. Overall, joining Cur to chemotherapy protocols seems a beneficial approach in cancer therapy; however, further research for developing drug delivery systems and determining treatment efficacy in clinical settings is demanded.

## Figures and Tables

**Table 1:** Curcumin in combination with chemotherapeutics in cancer therapy.

Cancer Type	Combinational Regimen	Study Type	Suggested Mechanisms	Outcome	Reference
Breast cancer	Curcumin (10 µM, dissolved in DMSO) + Doxorubicin (0–100 µM)	In vitro (MCF-7 and MDA-MB-231 cells)	- Suppressing the ATPase function of ABCB4	- Inversing doxorubicin resistance in cancer cells	[56]
	Curcumin (20 μM or 100 μg/kg, dissolved in DMSO) + Cisplatin (2 μg/mL or 2 mg/kg)	In vitro (MCF-7 cells) and in vivo (MCF-7 xenografted mice)	- Reducing FEN1 expression - Inducing apoptosis and inhibiting cell proliferation	- Inhibiting tumor growth and augmenting sensitivity to cisplatin	[57]
	Curcumin (10 μM) + 5-FU (10 μM)	In vitro (SK-BR-3 and MCF7)	- Downregulating NF-κB - Hindering Akt/PI3K and MAPK pathways	- Inducing a synergistic cytotoxic effect (58% versus 5-FU (21%) or curcumin (12%) alone	[41]
	Curcumin (1 mg/mL) and 5-FU (1 mg/mL)-loaded silk fibroin nanoparticles	In vitro (4T1 cells) and in vivo (4T1 xenografted mice)	- Provoking ROS generation - Inducing apoptosis	- Improving cytotoxicity (90%) compared to free drugs (~8%)	[42]
	Curcumin (30 µM, dissolved in DMSO 0.1%) + Paclitaxel (10 µM)	In vitro (MDA-MB-231 cells)	- Inducing PARP activation - Decreasing NF-κB, PCNA, and caspase-3 protein expression - Inducing apoptosis	- Inducing a significant level of toxicity (79.9%) in comparison to monotherapy	[43]
	Curcumin (7.5 µg/mL) and GANT61 (12.5 µg/mL)-loaded PLGA nanoparticles	In vitro (MCF-7 cells)	- Reducing GLI1 and BMI-1 expression - Downregulating EGFR and PI3K signaling - Compromising the self-renewal capacity of CSCs - Inducing apoptosis and necrosis	- Inhibiting tumor growth - Exhibiting a viable strategy to eradicate the intra-heterogeneous breast tumor comprising of CSCs	[44]
Colorectal cancer	Curcumin (5 µM) + Irinotecan (100 µM)	In vitro (LoVo cells)	- Inhibiting tumor sphere formation - Reducing the expression of CSC markers - Inducing apoptosis in sphere-forming cancer cells	- Attenuating chemoresistance to irinotecan	[58]
	Curcumin (5 µM, dissolved in DMSO) + 5-FU (1 µM)	In vitro (HCT116 cells)	- Repressing the NF-κB/PI3K/Src signaling - Enhancing the cleavage of pro-apoptotic proteins (e.g., caspases, PARP, and Bax) - Downregulating Bcl-xL and cyclin D1 proteins	- Providing a more effective treatment approach against chemoresistant cancer cells	[26]
	PGV-1 (10 µM, dissolved in DMSO) + 5-FU (1 mM)	In vitro (WiDr cells)	- Inhibiting NF-κB activation - Suppressing COX-2 expression	- Increasing the S-phase cell accumulation up to 94% - Promoting the cytotoxic impact of 5-FU on cancer cells	[29]
	Curcumin (50 mg/kg, dissolved in 0.1% DMSO) + Vincristine (2 mg/kg)	In vivo (HCT-8/VCR cells xenografted mice)	- Reducing the expression of MRP1 and P-gp	- Inhibiting xenograft growth	[30]
	Curcumin (5–40 5 μM) + 5-FU (1.39 μg/mL)	In vitro (HCT-116 cells)	- Restraining the TET1/NKD2/Wnt signaling and EMT	- Enhancing the inhibitory effects of 5-FU against cancer cell growth	[32]
	Curcumin (60 mg/kg, dissolved in PEG400: ethanol: D5W = 4:1:5) + Oxaliplatin (10 mg/kg)	In vivo (HCT116/OXA xenografted mice)	- Inhibits the TGF-β/Smad signaling - Inhibiting the expressions of p-p65 and Bcl-2 - Upregulating active-caspase-3	- Reversing oxaliplatin resistance and reducing tumor weight by more than 50%	[33]
	Curcumin (20 μM) + FOLFOX (50 μM 5-FU + 1.12 μM Oxaliplatin)	In vitro (HCT-116 or HT-29 cells)	- Reducing CSCs as indicated by reduced expression of CD44, CD166 markers - Decreasing EGFR expression - Reducing the ability to form anchorage-dependent colonies - Disintegrating colonospheres	- Exhibiting an effective approach to prevent the evolution of chemoresistance	[37]
	Curcumin (25 or 50 μM, dissolved in ethanol) + FOLFOX (50 μM 5-FU and 1.25 μM Oxaliplatin)	In vitro (HCT-116 or HT-29 cells)	- Preventing the EGFR, HER2, IGF-1R, and Akt activation - Reducing COX-2 and cyclin-D1 expression	- Conferring up to 78% growth inhibition in chemo-surviving cells - Decreasing the chance of relapse following chemotherapy	[38]
Prostate cancer	Curcumin (20 μM, dissolved in DMSO) + Docetaxel (10 nm)	In vitro (DU145 and PC3 cells)	- Inhibiting NF-κB activation - Downregulating PI3K, pAKT, COX-2, and RTKs - Enhancing p53 expression - Inducing apoptosis and inhibiting proliferation	- Increasing the efficacy of docetaxel in prostate cancer treatment	[48]
	Curcumin + Paclitaxel	In vitro (DU145 and PC3 cells)	- Reducing the expression of CD^44^ and P-gp - Upregulating miR-148a - Targeting MSK1/IRS1 axis	- Restoring paclitaxel sensitivity	[50]
	Curcumin (5–40 µM, dissolved in ethanol) + Metformin (0.4–12 mM)	In vitro (LNCaP cells)	- Diminishing the expression of mTOR and Bcl-2 - Upregulating Bax and PUMA - Inducing apoptosis	- Inducing a more profound effect in suppressing tumor cell growth (83%) compared to curcumin (68%) or metformin (52%) alone	[51]
Gastric cancer	Curcumin + Doxorubicin (5 μg/mL)	In vitro (AGS cells)	- Decreasing cell survival, spheroid formation, migration, and invasion - Downregulating Bcl-2 and upregulating Bax - Provoking apoptosis	- Exhibiting remarkable apoptotic effects (46%) compared to curcumin (21%) or doxorubicin (35%) alone	[59]
Neuroblastoma	Curcumin (10 and 20 μM, dissolved in DMSO) + Doxorubicin (5 μg/mL)	In vitro (SH-SY5Y cell)	- Downregulating MMP-2 and upregulating TIMP-1, p53, and p21 - Inducing apoptosis and inhibiting 3D tumor migration	- Elevating the fraction of doxorubicin-triggered apoptosis from 21.76% to 57.74%	[60]
Lung cancer	Curcumin (10 µM or 1 g/kg, dissolved in DMSO) + Gefitinib (5 µM or 100 mg/kg)	In vitro (H157 and H1299 cells) and in vivo (H157 and H1299 xenografted mice)	- Inactivating EGFR by repressing Sp1, thwarting the relationship between Sp1 and HDAC1 - Suppressing RTKs activity as well as ERK/MEK and Akt/S6K signaling - Inducing autophagic cell death and autophagy-mediated apoptosis	- Enhancing the suppressive impact of gefitinib on chemoresistant cells	[61]
	Curcumin (20 μM or 50 mg/kg) + Cisplatin (5 μg/mL or 2.5 mg/kg)	In vitro (A549 cells) and in vivo (A549 xenografted mice)	- Enhancing the binding of Sp1 to CTR1 and Sp1 promoters, influencing CTR1 expression - Inhibiting cell multiplication and promoting apoptosis - Repressing copper influx and enhancing platinum ion uptake in tumor in vivo	- Augmenting the sensitivity of cancer cells to cisplatin treatment	[62]
Laryngeal squamous cancer	Curcumin (10 μM, dissolved in DMSO) + Cisplatin (25 μM)	In vitro (Hep2 cells)	- Upregulating TRPM2 - Provoking lipid peroxidation and mitochondrial oxidative stress	- Improving the sensitivity of cancer cells to cisplatin	[63]
Bladder cancer	Curcumin (25 μM) + Gemcitabine (10 μM)	In vitro (T24-GCB cells)	- Upregulating cleaved-PARP - Inducing apoptosis	- Enhancing the cytotoxic effect, thereby reversing gemcitabine resistance	[64]
Oral cancers	Curcumin (40 µM, dissolved in DMSO) + Cetuximab (20 µg/mL)	In vitro (CAL 27 cells)	- Elevating caspases activities and induction of apoptosis - Reducing EGFR protein expression and inhibiting MAPK signaling - Downregulating pERK, JNK, and p38	- Synergizing the cetuximab impacts in eradicating cancer cells - Reducing the viability of cancer cells to 38.6% compared to 72.8% with cetuximab alone	[65]
Hepatocellular carcinoma	Curcumin (40 μM, dissolved in DMSO) + Celecoxib (100 μM)	In vitro (HepG2 cells)	- Diminishing the levels of Akt, NF-κB, PGE2, MDA, CD1, and VEGF - Inducing caspase-3 activation	- Augmenting the celecoxib-mediated anti-tumor effects - Repressing cell growth by 79%	[66]
Renal cell carcinoma	Curcumin (20 μM or 50 mg/kg) + PP242 (0.5 μM or 20 mg/kg)	In vitro (Caki cells) and in vivo (Caki cells xenografted mice)	- Provoking autophagy-mediated cell death - Downregulating Rictor and Akt	- Diminishing tumor growth and triggering cell death	[67]

ABCB4, also known as multi-drug resistance 3; EGFR, epidermal growth factor receptor; Sp1, a transcription factor; HDAC1, histone deacetylase 1; RTKs, receptor tyrosine kinases; ERK, extracellular signal-regulated kinase; FEN1, flap structure-specific endonuclease 1; CTR1, copper transporter 1; TRPM2, transient receptor potential melastatin 2; PARP, poly (ADP-ribose) polymerase; CSCs, cancer stem cells; MAPKs, mitogen-activated protein kinases; NF-κB, nuclear factor-κB; PGE2, prostaglandin E2; MDA, malondialdehyde; CD1, cluster of differentiation 1; VEGF, vascular endothelial growth factor; PP242, an mTOR1/2 inhibitor; DMSO, dimethyl sulfoxide; 5-FU, fluorouracil; PI3K, phosphatidylinositol-3-kinase; ROS, reactive oxygen species; PLGA, polylactic-co-glycolic acid; PEG, polyethylene glycol; PCNA, proliferating cell nuclear antigen; GLI1, glioma-associated oncogene homologue 1; BMI-1, B lymphoma Mo-MLV insertion region 1 homolog; COX-2, cyclooxygenase-2; MRP1, multidrug resistance-associated protein 1; P-gp, P-glycoprotein; TET1, ten-eleven translocation methylcytosine dioxygenase 1; NKD2, naked cuticle homolog 2; EMT, epithelial mesenchymal transition; TGF-β, transforming growth factor beta; IGF-1R, insulin-like growth factor 1 receptor; MSK1, mitogen- and stress-activated kinase 1; IRS1, insulin receptor substrate-1; mTOR, mammalian target of rapamycin; PUMA, p53 upregulated modulator of apoptosis; MMP-2, matrix metalloproteinase-2; TIMP-1, tissue inhibitor of metalloproteinase-1; JNK, c-Jun N-terminal kinases.

**Table 2:** Cur in combination with other phytochemicals in cancer therapy.

Type of Cancer	Combinational Regimen	Study Type	Suggested Mechanism	Outcome	Reference
Pancreatic cancer	Curcumin (5 μM or 2000 ppm dissolved in corn oil) + DHA (25 μM)	In vitro (BxPC-3 cells) and in vivo (BxPC-3 cells xenografted mice)	- Downregulating iNOS, COX-2, and 5-LOX - Upregulating p21 - Inducing apoptosis and inhibiting cell proliferation	- Reducing tumor volume by more than 72%	[89]
Colorectal cancer	Curcumin (0.5 ng/ul or 100 mg/kg, dissolved in DMSO) + Proanthocyanidins (25 ng/ul or 100 mg/kg)	In vitro (HCT116, SW480, SW620, HT29, RKO, and LoVo cells) and in vivo (HCT116 xenografted mice)	- Altering the expression of HSPA5, SEC61B, G6PD, HMOX1, and PDE3B genes - Affecting DNA replication and mismatch repair, cell cycle pathway, and GSH and porphyrin metabolism	- Offering superior anti-tumorigenic features	[90]
	Curcumin emulsome (25 μM) + Piperine emulsome (7 μM)	In vitro (HCT116 cells)	- Arresting cell cycle at G2/M phase - Repressing cell proliferation - Increasing caspase 3 level - Provoking apoptosis	- Improving the anti-cancer action of the compounds - Inhibiting cell proliferation of about 50% viability	[91]
	Curcumin (20.5 μM, dissolved in DMSO) + Resveratrol (51.3 μM)	In vitro (DLD-1 cells)	- Targeting PMAIP1, BID, ZMAT3, CASP3, CASP7, and FAS genes - Provoking apoptosis and repressing cell proliferation	- Exhibiting a synergistic anti-cancer effect	[92]
Breast cancer	Curcumin (10 μM, dissolved in DMSO) + Quercetin (20 μM)	In vitro (MDA-MB-231)	- Inhibiting cell viability and immigration - Inducing BRCA1 promoter histone acetylation	- Acting synergistically to produce anti-cancer activity - Conferring the cell survival rate of 0.38%	[93]
	Curcumin (24.5 μM, dissolved in DMSO) + Thymoquinone (51.76 μM)	In vitro (MDA-MB-231)	- Upregulating caspase-3 - Reducing PI3K and Akt protein levels - Provoking apoptosis and suppressing the cell cycle progression	- Exhibiting promising anti-cancer benefits - Inducing considerable apoptosis (75.76%)	[94]
	Curcumin (30 μM, dissolved in DMSO) + Gallic acid (50 μM)	In vitro (MDA-MB-231)	- Generating ROS - Depleting GSH storage - Decreasing Bcl-2 while increasing Bax, cleaved-caspase3, and PARP levels - Halting cell cycle inducing apoptosis	- Remarkably suppressing cell growth (64%) compared to gallic acid (23%) or curcumin (21%) - Providing a promising candidate for chemoprevention	[95]
	Curcumin (2.74 μM) and Quercetin (3 μM)-loaded Apoferritin nanoparticles	In vitro (MCF-7)	- Allowing cellular uptake of natural agents - Triggering intracellular ROS generation - Provoking apoptosis	- Enhancing the synergistic cytotoxic impact of phytochemicals by improving their bioavailability and effective targeting of cancer cells	[96]
	Curcumin and Chrysin-loaded PLGA-PEG nanoparticles (0.47 μM)	In vitro (MDA-MB-231)	- Upregulating miR-132 and miR-502c - Downregulating HN1 and P65 - Halting cell cycle at G2/M phase - Inducing apoptosis	- Significantly inducing apoptosis (60.1%) compared to non-formulated compounds (43.1%) - Demonstrating synergistic antiproliferative effects	[97]
Melanoma	Curcumin (2 µM, dissolved in DMSO) + Quercetin (2–8 µM)	In vitro (A375 cells)	- Downregulating Wnt/β-catenin signaling pathway proteins, e.g., β-catenin, cyclin D1, COX-2, and Axin2. - Downregulating Bcl-2 and inducing caspase-3/7 through PARP cleavage - Inhibiting cell proliferation and promoting apoptosis	- Synergistically inhibiting cancer cell growth	[98]
Chronic myeloid leukemia	Curcumin (21.43 mM, dissolved in DMSO) + Quercetin (84.02 mM)	In vitro (K562 cells)	- Increasing ROS and reducing GSH levels - Impairing mitochondrial membrane potential - Inducing cytochrome c release causing PARP and caspase-9 cleavage - Inducing apoptosis	- Potentiating apoptotic impacts of the compounds - Decreasing effective doses, thus, preventing toxic impacts on normal cells	[99]
Ovarian cancer	Curcumin (30 µM) + Resveratrol (70 µM)	In vitro (A2780 cells)	- Suppressing the PI3K/Akt/mTOR pathway	- Sensitizing cancer cells to cisplatin	[100]
Lung cancer	Curcumin + Paris Saponin II (0.125 to 2.0 μM)	In vitro (NCI-H1299, NCI-H460, NCI-H520, and NCI-H446 cells)	- Activating DR6, CD40/CD40L, FasL, and TNF-α - Upregulating IGFBP-1, resulting in the suppression of PI3K/Akt signaling - Upregulating p21 and p27 expression levels - Suppressing PCNA and NF-κB pathways - Inducing the phosphorylation of p38, JNK, and ERK - Arresting cell cycle and inducing apoptosis	- Exhibiting a synergistic anti-cancer effect	[101]

DHA, docosahexaenoic acid; HSPA5, heat Shock Protein Family A (Hsp70) Member 5; G6PD, glucose-6-phosphate dehydrogenase; HMOX1, heme oxygenase 1; PDE3B, phosphodiesterase 3B; BRCA1, breast cancer gene 1; iNOS, inducible nitric oxide synthase; 5-LOX, 5-lipoxygenase; COX-2, cyclooxygenase-2; Axin2, axis inhibition protein 2; PARP, poly (ADP-ribose) polymerase; DMSO, dimethyl sulfoxide; ROS, reactive oxygen species; GSH, glutathione; PI3K, phosphoinositide 3-kinase; mTOR, mammalian target of rapamycin; DR6, death receptor 6; FasL, Fas-ligand; TNF-α, tumor necrosis factor alpha; CD40, cluster of differentiation 40; CD40L, CD40-ligand; IGFBP-1, insulin-like growth factor-binding protein-1; p21and p27, potent cyclin-dependent kinase inhibitors; p65; one of the subunits of the NF-κB complex; PCNA, proliferating cell nuclear antigen; NF-κB, nuclear factor-κB; p38, a mitogen-activated protein kinase; JNK, c-Jun N-terminal kinase; HN1, haematological- and neurological-expressed 1.

## Data Availability

Not applicable.
